# Optical property dataset of inorganic phosphor

**DOI:** 10.1038/s41598-024-58351-w

**Published:** 2024-04-01

**Authors:** Seunghun Jang, Gyoung S. Na, Yunhee Choi, Hyunju Chang

**Affiliations:** Korea Research Institute of Chemical Technology (KRICT), Chemical Data-Driven Research Center, Daejeon, 34114 Republic of Korea

**Keywords:** Inorganic LEDs, Inorganic LEDs

## Abstract

Developing inorganic phosphor with desired properties for light-emitting diode application has traditionally relied on time-consuming and labor-intensive material development processes. Moreover, the results of material development research depend significantly on individual researchers’ intuition and experience. Thus, to improve the efficiency and reliability of materials discovery, machine learning has been widely applied to various materials science applications in recent years. However, the prediction capabilities of machine learning methods fundamentally depend on the quality of the training datasets. In this work, we constructed a high-quality and reliable dataset that contains experimentally validated inorganic phosphors and their optical properties, sourced from the literature on inorganic phosphors. Our dataset includes 3952 combinations of 21 dopant elements in 2238 host materials from 553 articles. The dataset provides material information, optical properties, measurement conditions for inorganic phosphors, and meta-information. Among the preliminary machine learning results, the essential properties of inorganic phosphors, such as maximum Photoluminescence (PL) emission wavelength and PL decay time, show overall satisfactory prediction performance with coefficient of determination ($R^2$) scores of 0.7 or more. We also confirmed that the measurement conditions significantly improved prediction performance.

## Introduction

Because the material properties of color-conversion inorganic phosphor essentially determine the suitable applications and effectiveness of white light-emitting diodes (LEDs), developing inorganic phosphor materials with desired levels of durability, energy conversion efficiency, and power consumption is crucial^1–5^. Inorganic phosphors are generally formed by substituting (doping) the luminescent center atoms, such as Eu^2+^ or Mn^2+^, in a host crystal structure^6–8^. These materials absorb some blue-light emission from the InGaN LED chip and re-emit light of a longer wavelength, resulting in white light including a wide range of visible wavelengths^9,10^. Conventional research aiming to develop novel inorganic phosphor materials has relied on time-consuming and labor-intensive trial-and-error experiments^10–12^. For this reason, comprehensive investigations regarding novel inorganic materials in unexplored material spaces require a large amount of resources and time.

Recently, the Brgoch group employed machine learning (ML) to predict the Debye temperature based on a dataset of the calculated bulk and shear moduli^13^. Using this ML approach, they discovered a novel inorganic phosphor of the desired Debye temperature and density functional theory (DFT) band gap. However, the applicability of existing inorganic phosphor datasets is limited in real-world applications for two reasons. First, most existing datasets were constructed by collecting the calculated properties of the materials, which inevitably contain errors resulting from the calculation methods. Second, the existing datasets only contain limited types of physical properties of inorganic phosphor materials.

A well-refined training dataset is crucial for successful ML in data-driven materials science. However, constructing a large dataset containing various materials requires substantial time and effort to generate experimental observations because time-consuming and labor-intensive material synthesis and property measurement experiments must be conducted. Although theoretical approaches could be used instead, the practicality of creating a theoretical dataset through calculation methods such as DFT is also limited owing to the high computational costs of the calculation methods. An efficient alternative to constructing a large materials dataset is collecting data from the literature^13–15^. Although data collected from the literature can be biased toward several material groups, materials datasets collected from literature searches are attractive in ML for materials science because they can provide many experimental observations on the target materials.

For data-driven research on inorganic phosphors, we conducted a literature search to construct a materials dataset that contains experimentally synthesized inorganic phosphors and their experimentally measured physical properties. We refer to this inorganic phosphor optical property dataset as the IPOP dataset^16^. We collected eight essential physical properties of inorganic phosphors, such as photoluminescence (PL) maximum wavelengths, Commission Internationale de I’Eclairage (CIE) coordinates, lifetime, and quantum efficiency. In addition to the physical properties, we also collected the measurement conditions to ensure the reliability of the dataset. The IPOP dataset contains 16,023 observations of 2238 host materials^16^. To the best of our knowledge, this dataset is the largest public dataset of inorganic phosphors. The IPOP dataset can be used for data-driven discovery of novel inorganic phosphors beyond a simple dataset search by employing high-throughput screening based on the prediction models trained on the dataset. This high-throughput screening can also be conducted on general-purpose datasets that contain large amount of inorganic materials, such as Pearson’s Crystal dataset^17^. In addition to dataset construction, we conducted an ML experiment using the IPOP dataset to obtain preliminary ML results. In the prediction results part, we will briefly discuss the ML results on our inorganic phosphor dataset.

## Results and discussion

### Inorganic phosphor data collection

We collected the data for the inorganic phosphor dataset from 553 articles on inorganic phosphors directly downloaded from Springer Nature, American Chemical Society, Royal Society of Chemistry, Wiley, Elsevier, and IOPscience (Supplementary Fig. S1). We searched for articles using keywords such as “PL”, “PL excitation”, “decay time”, “quantum efficiency”, and “thermal quenching temperature”. datasets were collected mainly to collect quantitative data regarding the optical properties of inorganic phosphors. Our dataset comprises composition information and optical property information on a total of 3,952 inorganic phosphors, and the inorganic phosphor dataset was limited to cases involving up to two dopants (first and second dopants).

**Figure 1:**
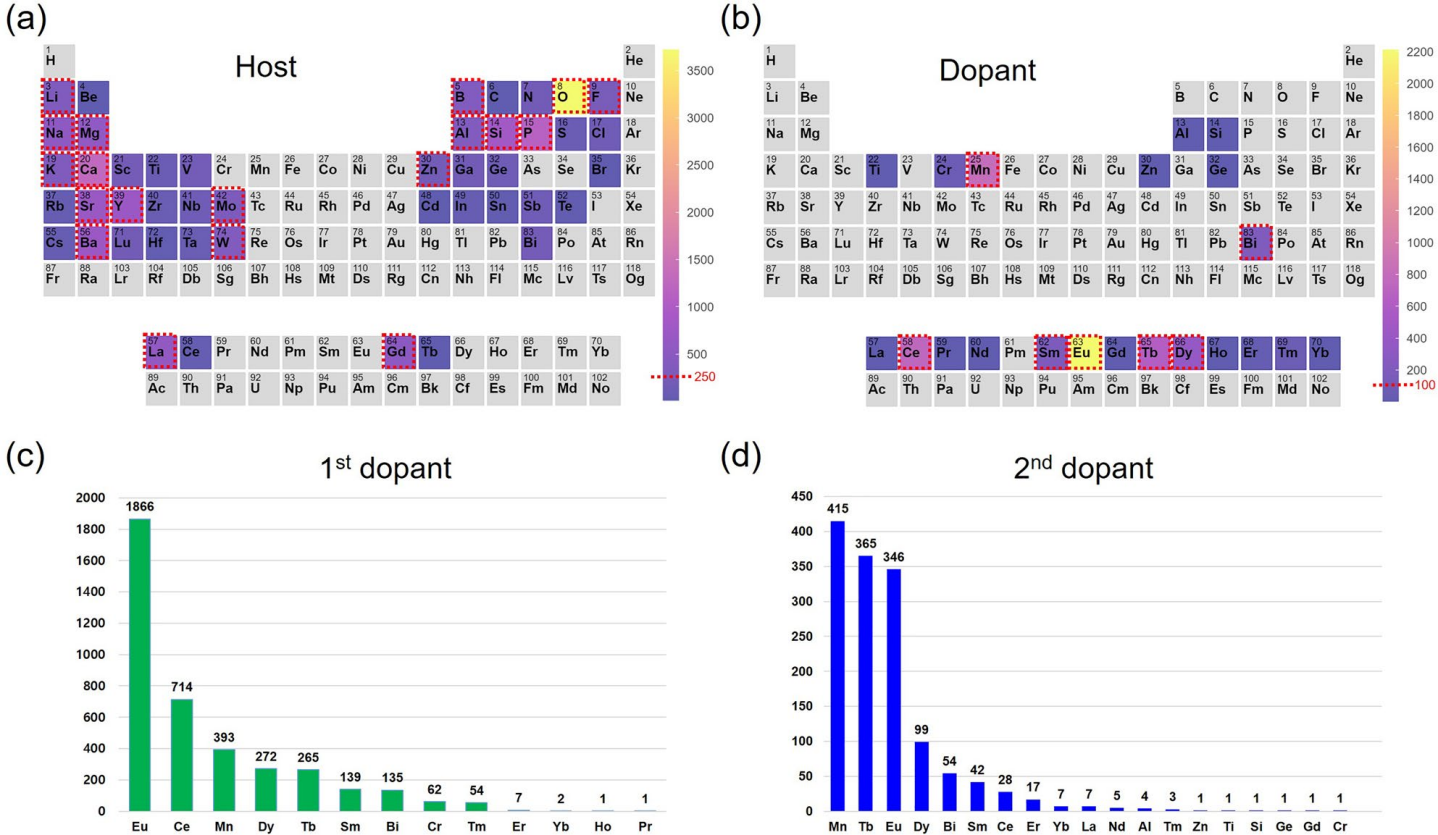
Element frequencies for the (**a**) hosts and (**b**) dopants of the inorganic phosphors in the dataset. Histograms of the elements comprising the (**c**) first and (**d**) second dopants.

Figure 1a, b shows the element frequencies for the hosts and dopants of the collected inorganic phosphor dataset. The dataset mainly contains host materials such as oxide, phosphide, boride, silicate, and fluoride. Furthermore, our dataset’s host materials include alkali metals such as Li, Na, and K, alkaline earth metals such as Mg and Ca, and transition metals such as Y, Mo, W, and Zn. As for dopant elements, the dataset mainly includes rare-earth elements (REEs) such as Eu, Sm, Tb, and Dy and elements such as Mn and Bi. In addition, transition metals are rarely included. In total, our dataset contains 21 types of dopant elements. Among the first dopants, Eu appears most frequently, as shown in Fig. 1c. More than half of the first dopants of the inorganic phosphors use Eu. In addition to Eu, elements such as Ce, Mn, Dy, and Tb are utilized as first dopants in many cases (more than 200). Additionally, the information about second dopants of inorganic phosphors is also shown in Fig. 1d.

**Figure 2:**
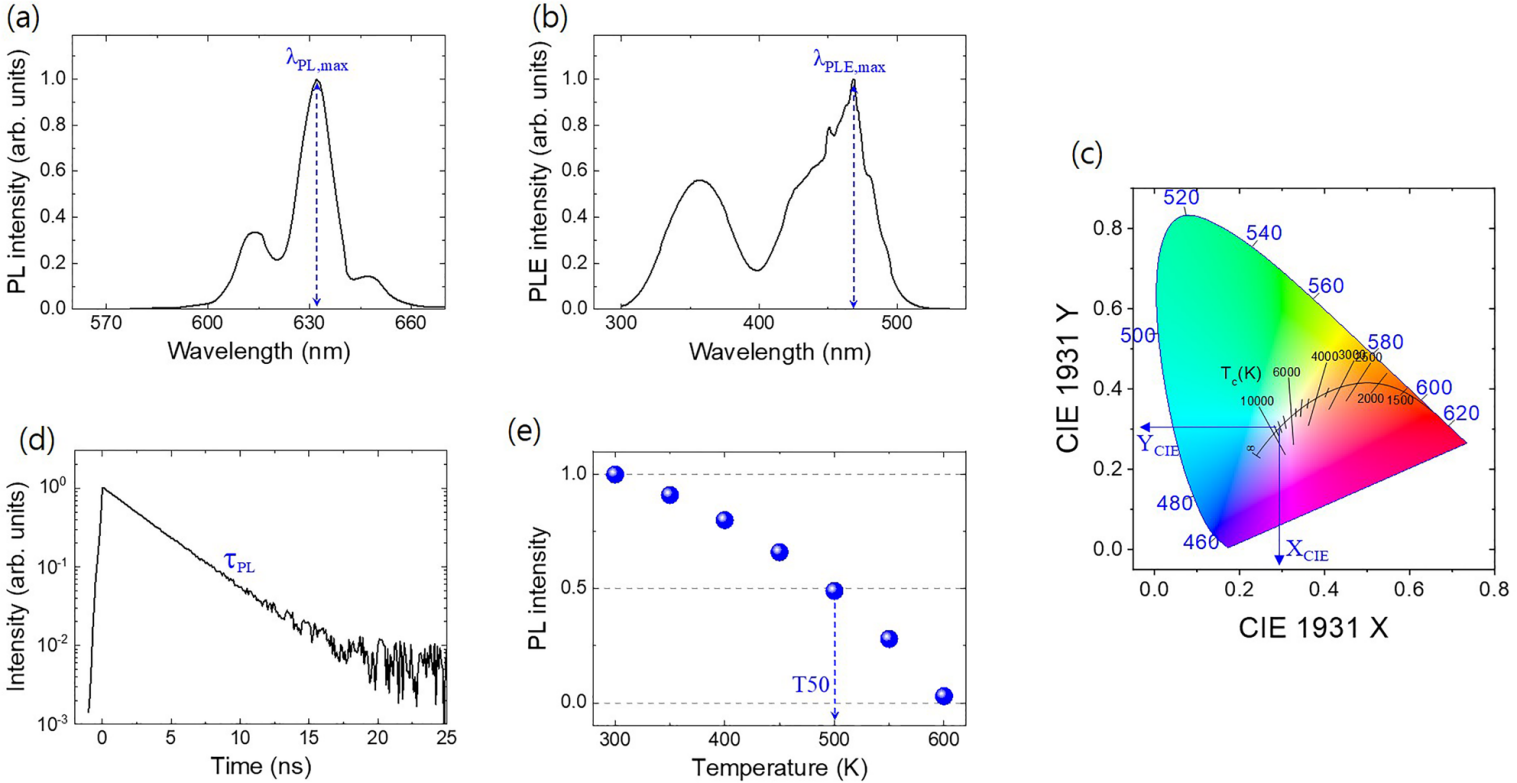
(**a**) Maximum photoluminescence (PL) emission wavelength, $\lambda _{PL,max}$, (**b**) maximum PL excitation wavelength, $\lambda _{PLE,max}$, (**c**) X and Y coordinates ($X_{CIE}$ and $Y_{CIE}$) in CIE chromaticity diagram, (**d**) PL decay time, $\tau _{PL}$, and (**e**) thermal quenching temperature, T50.

As illustrated in Fig. 2, we collected the optical and thermal stability properties for inorganic phosphor based on figures, tables, or specific quantitative values mentioned in the original articles. To simplify the process of collecting quantitative values from spectral data, we included only the peak positions ($\lambda _{PL,max}$) with the maximum intensities of PL in the data. (Fig. 2a) Considering the importance of PL excitation (PLE) data, these data were collected up to the third maxima ($\lambda _{PLE,max(1st)}$, $\lambda _{PLE,max(2nd)}$, $\lambda _{PLE,max(3rd)}$), whereas Fig. 2b only shows the first maxima of the PLE data. Distribution information for $\lambda _{PLE,max(2nd)}$ and $\lambda _{PLE,max(3rd)}$ can be confirmed by consulting Supplementary, Fig. S2. We also included temperature conditions of the PL and PLE measurements as fundamental parameters in the dataset and intentionally excluded optical properties measured below room temperature (RT) to prioritize the development of novel inorganic phosphor materials that work well at RT and above and can be directly applied industrially. In addition, our dataset provides the X and Y coordinates ($X_{CIE}$ and $Y_{CIE}$) in the CIE chromaticity diagram, which are crucial characteristics for determining the applications of inorganic phosphors. By applying ML using the collected CIE coordinates dataset, we can estimate what color light will be emitted using only simple information about the inorganic phosphor (e.g., composition).

Furthermore, we collected data on the properties of inorganic phosphors that were difficult to manage owing to the sparsity of the data, such as PL decay time ($\tau _{PL}$), quantum efficiency ($QE_{int}$ for internal, $QE_{ext}$ for external), and thermal quenching temperature (T50). For the PL decay time ($\tau _{PL}$), we collected the lifetime (from an excited state to a ground state) of electrons measured by time-resolved PL (TRPL) experiments. The PL decay data were typically fitted using the multi-exponential function $I(t) = \Sigma _{i} I_{i}$ $exp(-t/\tau _{i})$, where $I_{i}$ and $\tau _{i}$ are the amplitude and time constant, respectively. In each case, we took the component that contributed the most to the fitting among multiple time constants and included it in our dataset. External quantum efficiency ($QE_{ext}$) is an industrially valuable property, and internal quantum efficiency ($QE_{int}$) can also be utilized as an essential material characteristic. We included these two parameters separately in the dataset. Furthermore, we collected thermal quenching temperature data for industrial use. Therefore, we excluded papers that reported T50 values below RT by performing PL measurement experiments at cryogenic temperatures.

**Table 1:** Description of the inorganic phosphor dataset.

No.	Column name	Unit	Data type	Description
1	Tag	–	Float	The numbering of data points
2	Inorganic phosphor	–	String	Composition of phosphor
3	Host	–	String	Host composition of phosphor
4	$1\textrm{st}$ dopant	–	String	$1\textrm{st}$ dopant atom of phosphor
5	$1\textrm{st}$ dopant valency	–	Float	Valency of $1\textrm{st}$ dopant atom
6	$1\textrm{st}$ dopant concentration	–	Float	Doping concentration of 1*st* dopant
7	$2\textrm{nd}$ dopant	–	String	$2\textrm{nd}$ dopant atom of phosphor
8	$2\textrm{nd}$ dopant valency	–	Float	Valency of $2\textrm{nd}$ dopant atom
9	$2\textrm{nd}$ dopant concentration	–	Float	Doping concentration of $2\textrm{nd}$ dopant
10	Temp. (K)	K	Float	Measurement temperature
11	Emission max. (nm)	nm	Float	Maximum PL emission wavelength, $\lambda _{PL,max}$
12	CIE X coordinate	–	Float	X coordinate in CIE chromaticity diagram, $X_{CIE}$
13	CIE Y coordinate	–	Float	Y coordinate in CIE chromaticity diagram, $Y_{CIE}$
14	Int. quantum efficiency (%)	–	Float	Internal quantum efficiency, $QE_{int}$
15	Ext. quantum efficiency (%)	–	Float	External quantum efficiency, $QE_{ext}$
16	Thermal quenching temp. (K)	K	Float	Thermal quenching temperature, T50
17	Excitation source (nm)	nm	Float	Wavelength of excitation source, $\lambda _{exc}$
18	1st excitation max. (nm)	nm	Float	1st Maximum PLE wavelength, $\lambda _{PLE,max(1st)}$
19	2nd excitation max. (nm)	nm	Float	2nd Maximum PLE wavelength, $\lambda _{PLE,max(2nd)}$
20	3rd excitation max. (nm)	nm	Float	3rd Maximum PLE wavelength, $\lambda _{PLE,max(3rd)}$
21	$Log_{10}$ Decay time ($log_{10}$ [ns])	$log_{10}$[ns]	Float	Logarithmic value of PL decay time,$\tau _{log,PL}$
22	Decay time (ns)	ns	Float	Photoluminescence decay time, $\tau _{PL}$
23	Monitoring energy (nm)	nm	Float	Decay time and PLE monitoring wavelength, $\lambda _{mon}$
24	Reference	–	String	Source document DOI
25	Publisher	–	String	Abbreviation for journal publisher
26	MP-ID	–	String	Materials project ID
27	ICSD-ID	–	String	Inorganic crystal structure database ID
28	LOF-Score	–	Float	Local outlier factor score

**Table 2:** Optical properties of Lu_3_Al_4.994_O_12_Mn_0.003_Mg_0.003_.

Tag	745
Inorganic phosphor	Lu_3_Al_4.994_O_12_Mn_0.003_Mg_0.003_
Host	Lu_3_Al_4.994_O_12_
$1\textrm{st}$ dopant	Mn
$1\textrm{st}$ dopant valency	4
$1\textrm{st}$ dopant concentration	0.003
$2\textrm{nd}$ dopant	Mg
$2\textrm{nd}$ dopant valency	2
$2\textrm{nd}$ dopant concentration	0.003
Temp. (K)	298
Emission max. (nm)	670
CIE x coordinate	0.713
CIE y coordinate	0.286
Int. quantum efficiency (%)	72.41
Ext. quantum efficiency (%)	41.39
Thermal quenching temp. (K)	NaN
Excitation source (nm)	326
Excitation max. (nm)	326
$Log_{10}$ Decay time ($log_{10}$ [ns])	6.094122
Decay time (ns)	1242000
Monitoring energy (nm)	670
Reference	https://doi.org/10.1039/C7TC02514A
Publisher	RSC
MP-ID	mp-14132
ICSD-ID	icsd-182354, icsd-23846
LOF-Score	0.971190219

Our dataset comprises 3952 combinations of 21 dopant atoms in 2238 host materials. The dataset contains four types of data: (1) material information of the host material and dopant element; (2) measurement conditions such as wavelength of excitation and monitoring energy; (3) target properties of inorganic phosphors including maximum emission position, decay time, and quantum efficiency; and (4) meta-information such as source document digital object identifier (DOI), tag number, local outlier factor (LOF) score, materials project (MP) ID, and inorganic crystal structure database (ICSD) ID. The format of the inorganic phosphor dataset is described in Table 1. Material information and measurement conditions correspond to input information, and target physical properties correspond to output information. Furthermore, meta-information (MP-ID ,ICSD-ID) on the structure that best matches the original composition of each host material is included in our dataset. Using the link information for the material structure, we can download the structure file for the corresponding host material in the MP and perform ML using crystal structure dataset, such as crystal graph convolutional neural networks^18^. Additionally, users can conduct data research by referring to the outlier factor (LOF-Score) value and determining whether the data are unnecessary. A data example of Lu_3_Al_4.994_O_12_Mn_0.003_Mg_0.003_ is presented in Table 2. Data that are not reported in the literature, such as thermal quenching temperature, are indicated as NaN (not a number).

### Dataset statistical analysis

We aim to provide industry and academia with a high-quality and trustworthy dataset of the optical properties of inorganic phosphors. Therefore, we collected data from 549 published papers that were cited more than 10 times, excluding self-citations, as of March 21, 2024 (Only 4 papers were cited less than 10 times) using the following procedure. Two researchers with sufficient background in materials and measurement of optical properties of inorganic phosphors designed the dataset through discussion and consultation. Afterward, one researcher focused on collecting the data, while the other focused on validating and cross-checking the collected dataset.

**Figure 3:**
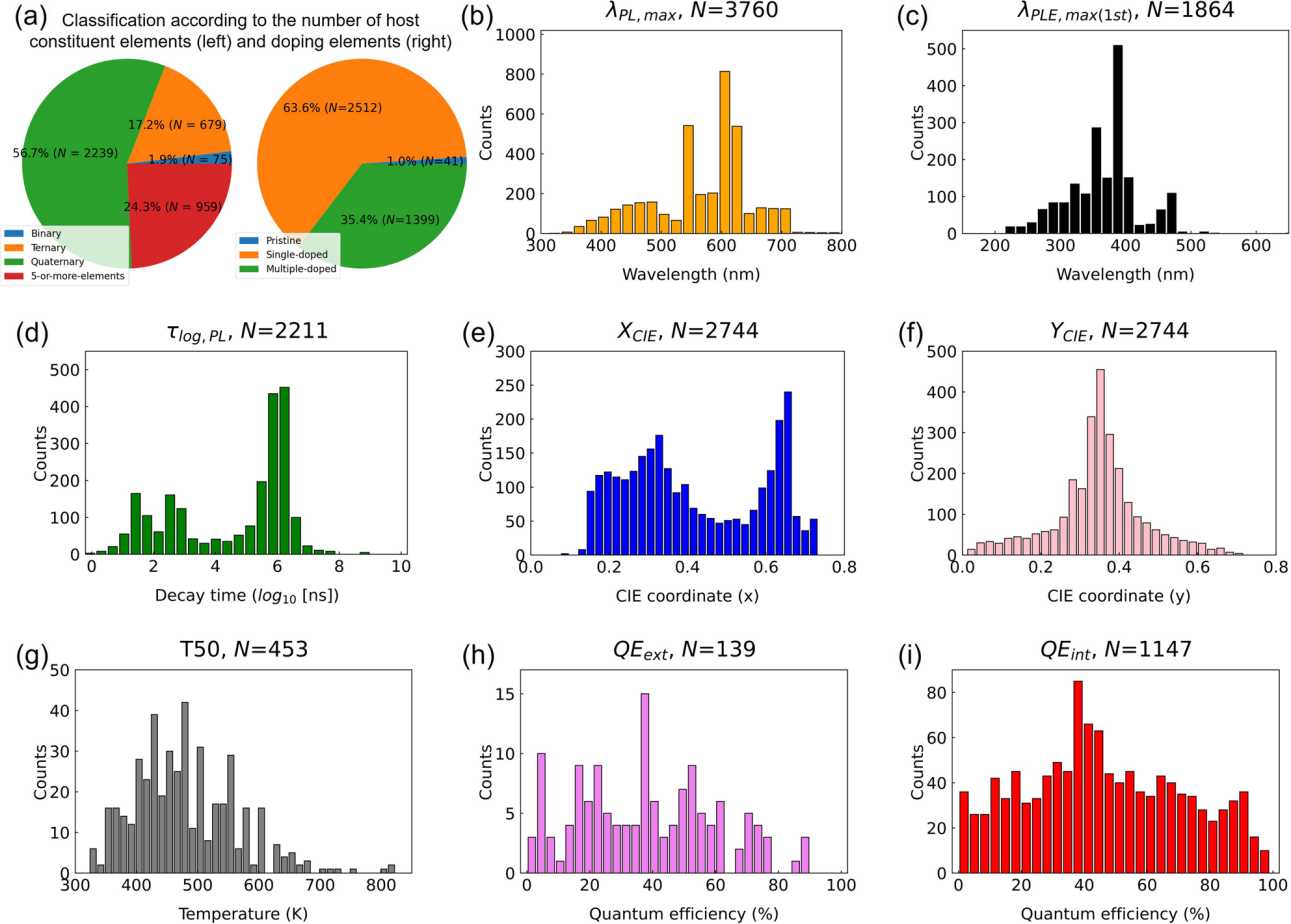
Distribution of the material and optical properties of the inorganic phosphors: number of constituent elements of the (a) host (left) and dopant (right), (b) $\lambda _{PL,max}$, (c) $\lambda _{PLE,max(1st)}$, (d) $\tau _{log,PL}$, (e) $X_{CIE}$, (f) $Y_{CIE}$, (g) T50, (h) $QE_{ext}$, and (i) $QE_{int}$. “Counts” on the y-axis refer to the number of collected data.

Figure 3 presents an overall summary of the final collected dataset. As shown in Fig. 3a, among the 3952 inorganic phosphors we collected, only 1.9% were binary compound hosts, 17.2% were ternary compounds, 56.7% were quaternary compounds, and the remaining 24.3% were hosts with five or more elements. Quaternary compounds accounted for the most significant proportion, which means that most inorganic phosphors currently being studied have complex host structures composed of four or more elements. We also analyzed the distribution of inorganic phosphors according to the number of dopants. We found that 63.6% of cases were single-doped, and the remaining cases (35.4%) were doped with two elements. This shows that studies using multiple dopants to improve the properties of inorganic phosphors are being actively conducted.

Figure 3b, c shows the histograms of $\lambda _{PL,max}$ and $\lambda _{PLE,max(1st)}$. Most of the $\lambda _{PL,max}$ is located in the region of visible light emission (380–700 nm). Two peaks near 540 nm and 620–640 nm indicate that research on green and red conversion phosphors is commonly conducted. The PLE of phosphors with a visible emission range mainly occurs in around 400 nm region in Fig. 3c, which is a region that overlaps somewhat with the emission wavelength of the typical InGaN-based LED (around 400–450 nm). It is difficult to efficiently visualize the data distribution of decay time, $\tau _{PL}$, if original values are used. Thus, we analyzed the distribution of the logarithmic decay time, $log_{10} \tau _{PL}$ values, as shown in Fig. 3d. Each peak can be identified in the $\tau _{PL}$ region with sub-$\mu {s}$, $\mu {s}$, and *ms* time scales. Interestingly, the $\tau _{PL}$ does not have continuous values; instead, the values are clustered and distributed in a specific area. The CIE chromaticity coordinates calculated from the emission spectra of phosphors were separated into $X_{CIE}$ and $Y_{CIE}$, shown in Fig. 3e, f. $Y_{CIE}$ is mainly concentrated between 0.3 and 0.4, whereas $X_{CIE}$ is distributed over a relatively wide area. Figure 3g displays the temperature-dependent distribution of T50 values extracted from temperature-dependent emission spectra. To experimentally obtain the value of T50, conducting PL measure experiments at various temperatures is necessary. Thus, T50 values are not expected to be widely reported in the literature. Nevertheless, we collected and provided 300 T50 data points in this study. About 80% of the T50 values are distributed between 400 K and 600 K. The external and internal quantum efficiencies, such as thermal quenching temperature, have small data points but are industrially meaningful values. Our dataset contains 157 $QE_{ext}$ and 1168 $QE_{int}$ data points reported in % units, as shown in Fig. 3h, i. The target properties of the inorganic phosphor mentioned above include the measurement conditions for each target property. Connection information between the target characteristics and measurement conditions is provided in Supplementary, Table S1. For example, to accurately specify $\lambda _{PL,max}$, Temp. and $\lambda _{exc}$ must be presented together.

### Prediction results for optical properties of inorganic phosphors

To experiment with the usability of the dataset we collected, we preliminarily predicted the optical properties of inorganic phosphors using extreme gradient boosting tree regression (XGB), which has shown state-of-the-art prediction accuracy in the field of material science^19^. The XGB algorithm shows excellent predictive performance even for a small number of datasets. To perform the prediction, we used the ML platform, SimPL-ML^20^. This platform is suitable for performing the ML tasks we envisioned because it provides atomic feature auto-generation, hyper-parameter optimization, and input feature selection. Because each activator has different structure-property relationships that affect the observed luminescence, we built prediction models using only the proprietary dataset for Eu activator rather than the entire IPOP dataset. To ensure that minimum data is required to build the prediction models, we used a dataset containing all Eu activators without distinguishing between Eu^2+^ and Eu^3+^ activators. If the user wants to generate optical property prediction models for activators other than Eu, the user must create a sub-dataset containing only the desired activator through the sorting function. However, users should keep in mind that if the number of sub-dataset is too small, the performance of the prediction model cannot be guaranteed. For information on the number of data for each activator ion, refer to Fig. 1c, d. Besides, users who want to generate additional atomic attribute information based on the chemical formula of the inorganic phosphor can visit the SimPL-ML platform and use the atomic feature auto-generation function.

**Figure 4:**
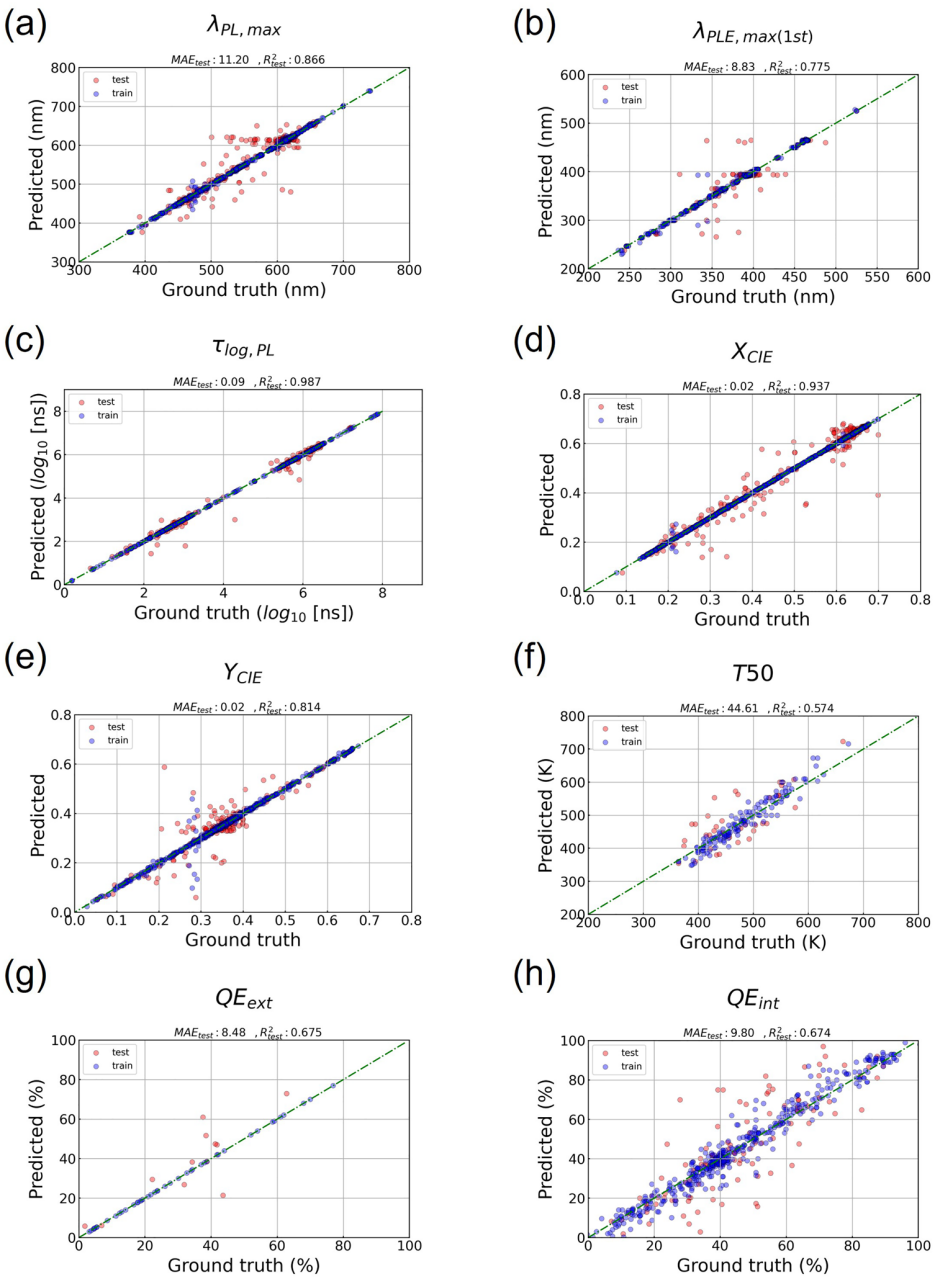
Prediction results by the XGB methods on the proprietary dataset of the Eu activator.

First, we converted the chemical formulas of inorganic phosphors into machine-readable feature vectors based on elemental attributes of the elements in the chemical formulas by using the atomic feature auto-generation of SimPL-ML. A total of 52 features were created using the atomic ratio, atomic number, atomic weight, atomic radius, Pauling electronegativity, number of valence electrons, first ionization energy, and the combination thereof. To allow anyone to freely utilize the intermediate results created in this process, we provide machine-readable feature vectors for inorganic phosphors for download in addition to the IPOP dataset^16^. These eight CSV (comma separated values) formatted text files containing atomic attribute information based on atomic formulas can be downloaded from https://doi.org/10.6084/m9.figshare.24771186. In addition to the atomic features (AF), we used measurement conditions (i.e., Temp., $\lambda _{exc}$, and $\lambda _{PLE,dec}$) as the input of the prediction models. For each set of chemical formulas and measurement conditions used as input, we predicted eight physical properties of the inorganic phosphors: $\lambda _{PL,max}$, $\lambda _{PLE,max(1st)}$, $\tau _{log,PL}$, $X_{CIE}$, $Y_{CIE}$, T50, $QE_{ext}$, and $QE_{int}$, as shown in Fig. 4. Additionally, the prediction results for $\lambda _{PLE,max(2nd)}$ can be confirmed by consulting Supplementary Fig. S3.

The prediction results for $\lambda _{PL,max}$, $\lambda _{PLE,max(1st)}$, $\tau _{log,PL}$, $X_{CIE}$, and $Y_{CIE}$ show overall satisfactory prediction performance with coefficient of determination ($R^2$) scores of 0.7 or more, but in the case of predictions of $QE_{ext}$, $QE_{int}$, and T50, with less than 800 data points, the $R^2$ scores were less than 0.69^21^. Additionally, we performed property prediction by varying the feature combinations to confirm that the Temp. (T), $\lambda _{exc}$ (ES), and $\lambda _{mon}$ (ME) values are necessary to accurately predict the optical properties of various inorganic phosphors. The prediction performance results for various feature combinations are shown in Table 3. In all predictions, a clear prediction performance improvement was confirmed by adding the ES input feature. Then, for the $\lambda _{PLE,max(1st)}$ and $\tau _{log,PL}$ predictions, we confirmed that the ME measurement condition feature is a crucial input feature for more accurate prediction. Contrary to our expectations, we could not confirm any prediction performance improvement according to the T input feature in all predictions, except for the $\lambda _{PLE,max(1st)}$ and $\tau _{log,PL}$ predictions.

To better understand the cause for this, we analyzed the data distribution for the T input features. As a result, for the $\lambda _{PL,max}$ target property, it was found that 93.2% of the T values were distributed at 293 K (RT). Accordingly, we constructed a subset of $\lambda _{PL,max}$ in which the proportion of $\lambda _{PL,max}$ measured under RT measurement conditions was reduced to 62.8%. Surprisingly, in the subset of the $\lambda _{PL,max}$ dataset, target prediction performance improved when the T input feature was added. Therefore, we conclude that the experimental measurement conditions such as T, ES, and ME provide a greater contribution to predicting various optical properties of inorganic phosphor through continuous data expansion and supplementation. Apart from this study, we plan to supplement the incomplete parts of the IPOP dataset through additional data expansion and supplementation.

Recently, various groups have reported experimental physical property prediction using a chemical formula-based feature alone^22–27^. In addition to chemical formula-based features, if additional information related to physical property measurements is used as a feature to generate a prediction model, further improvement of the prediction model can be expected. This means that beyond property predictors, the property measurement of virtual materials is also feasible.

Furthermore, we analyzed the correlation between the targets of the inorganic phosphor dataset (Supplementary, Fig. S4). Most of the target pairs have Pearson correlation values less than 0.5. Overall, because the relationship between the targets is independent, each target has potential for utilization. Among them, $\lambda _{PL,max}$ and $X_{CIE}$ show a Pearson correlation coefficient of 0.74. This result is interesting, considering that $\lambda _{PL,max}$ and $Y_{CIE}$ have a value of 0.35. We can find clues to understand this phenomenon in Fig. 2c. $X_{CIE}$ expresses a much more dramatic color change from red to blue than $Y_{CIE}$. Therefore, $X_{CIE}$ is more suitable for expressing its distributed information by following the PL peak position($\lambda _{PL,max}$) of inorganic phosphor. Additionally, the coefficient between $X_{CIE}$ and $\tau _{log,PL}$ is 0.64, which is generally understood to be mainly related to the longer PL decay time as the emission wavelength becomes longer in inorganic phosphor^28^.

**Table 3:** Results of the prediction of the properties of an inorganic phosphor according to various feature combinations.

Target property	Feature	XGB		
		MAE	RMSE	R^2^
$\lambda _{PL,max}$	AF+T+ES	14.611 ± 1.438	30.672 ± 1.469	0.760 ± 0.022
	AF+ES	15.098 ± 1.904	32.736 ± 3.399	0.724 ± 0.053
	AF+T	17.676 ± 0.791	36.341 ± 1.925	0.661 ± 0.039
	AF	17.665 ± 1.273	36.377 ± 2.150	0.666 ± 0.039
Subset of $\lambda _{PL,max}$	AF+T+ES	4.546 ± 2.504	13.222 ± 7.509	0.894 ± 0.101
	AF+ES	4.409 ± 2.035	15.693 ± 10.184	0.788 ± 0.197
	AF+T	6.498 ± 4.870	12.796 ± 9.532	0.861 ± 0.144
	AF	7.899 ± 2.935	23.562 ± 9.891	0.736 ± 0.195
$\lambda _{PLE,max(1st)}$	AF+T+ME	12.139 ± 1.412	25.863 ± 2.440	0.694 ± 0.043
	AF+ME	11.534 ± 1.278	25.358 ± 1.891	0.703 ± 0.026
	AF+T	13.017 ± 1.130	26.448 ± 2.228	0.680 ± 0.047
	AF	14.657 ± 1.171	28.507 ± 1.455	0.642 ± 0.034
$\tau _{log,PL}$	AF+ES+ME+T	0.150 ± 0.006	0.389 ± 0.036	0.944 ± 0.012
	AF+ES+ME	0.175 ± 0.035	0.447 ± 0.083	0.927 ± 0.026
	AF+T+ES	0.258 ± 0.022	0.547 ± 0.053	0.897 ± 0.020
	AF+T+ME	0.177 ± 0.019	0.452 ± 0.087	0.926 ± 0.026
	AF+ME	0.175 ± 0.020	0.442 ± 0.066	0.927 ± 0.023
	AF+ES	0.306 ± 0.042	0.585 ± 0.045	0.885 ± 0.016
	AF+T	0.339 ± 0.029	0.726 ± 0.059	0.820 ± 0.030
	AF	0.401 ± 0.049	0.787 ± 0.088	0.790 ± 0.047
$X_{CIE}$	AF+T+ES	0.037 ± 0.003	0.062 ± 0.003	0.870 ± 0.015
	AF+ES	0.036 ± 0.002	0.063 ± 0.002	0.867 ± 0.013
	AF+T	0.053 ± 0.001	0.092 ± 0.003	0.718 ± 0.020
	AF	0.050 ± 0.001	0.084 ± 0.004	0.763 ± 0.024
$Y_{CIE}$	AF+T+ES	0.029 ± 0.002	0.056 ± 0.002	0.696 ± 0.024
	AF+ES	0.027 ± 0.002	0.052 ± 0.005	0.729 ± 0.041
	AF+T	0.033 ± 0.003	0.060 ± 0.003	0.653 ± 0.028
	AF	0.033 ± 0.004	0.059 ± 0.003	0.668 ± 0.036
T50	AF+ES	46.152 ± 5.379	58.394 ± 6.810	0.445 ± 0.121
	AF	25.980 ± 4.212	41.450 ± 5.863	0.302 ± 0.084
$QE_{ext}$	AF+T+ES	9.056 ± 1.321	12.476 ± 2.474	0.499 ± 0.225
	AF+ES	9.476 ± 1.880	12.107 ± 2.627	0.529 ± 0.142
	AF+T	9.538 ± 2.552	11.777 ± 3.086	0.452 ± 0.221
	AF	9.683 ± 3.330	13.559 ± 3.476	0.476 ± 0.240
$QE_{int}$	AF+T+ES	9.368 ± 0.977	14.430 ± 1.388	0.635 ± 0.093
	AF+ES	9.152 ± 0.544	13.746 ± 1.254	0.652 ± 0.086
	AF+T	9.559 ± 0.797	14.659 ± 1.076	0.607 ± 0.075
	AF	9.962 ± 0.699	14.612 ± 0.890	0.614 ± 0.089

### Supplementary Information

Supplementary Information.

## Data Availability

This dataset is available at https://doi.org/10.6084/m9.figshare.24771186 (or https://github.com/KRICT-DATA/IPOP-dataset-ver-3.0). In addition, the CSV formatted text files containing atomic attribute information based on atomic formulas, which were created at www.simpl-ml.org, are also available.
