# Supplementary material for: Optical property dataset of inorganic phosphor
Source: Sci Rep. 2024 Apr 1;14:7639. doi: 10.1038/s41598-024-58351-w (PMC10984968; doi:10.1038/s41598-024-58351-w)
Supplement: Supplementary file 1 — Supplementary Information. [file 41598_2024_58351_MOESM1_ESM.pdf]

Optical property dataset of inorganic phosphor

Seunghun Jang[1,*], Gyoung S. Na[1], Yunhee choi[1], and Hyunju Chang[1,*]

[1]Korea Research Institute of Chemical Technology (KRICT), Chemical Data-Driven Research Center, Daejeon, 34114, Republic of Korea
[*]corresponding authors: Hyunju Chang (hjchang@krict.re.kr), Seunghun Jang (jang@krict.re.kr)

ABSTRACT

Supporting information

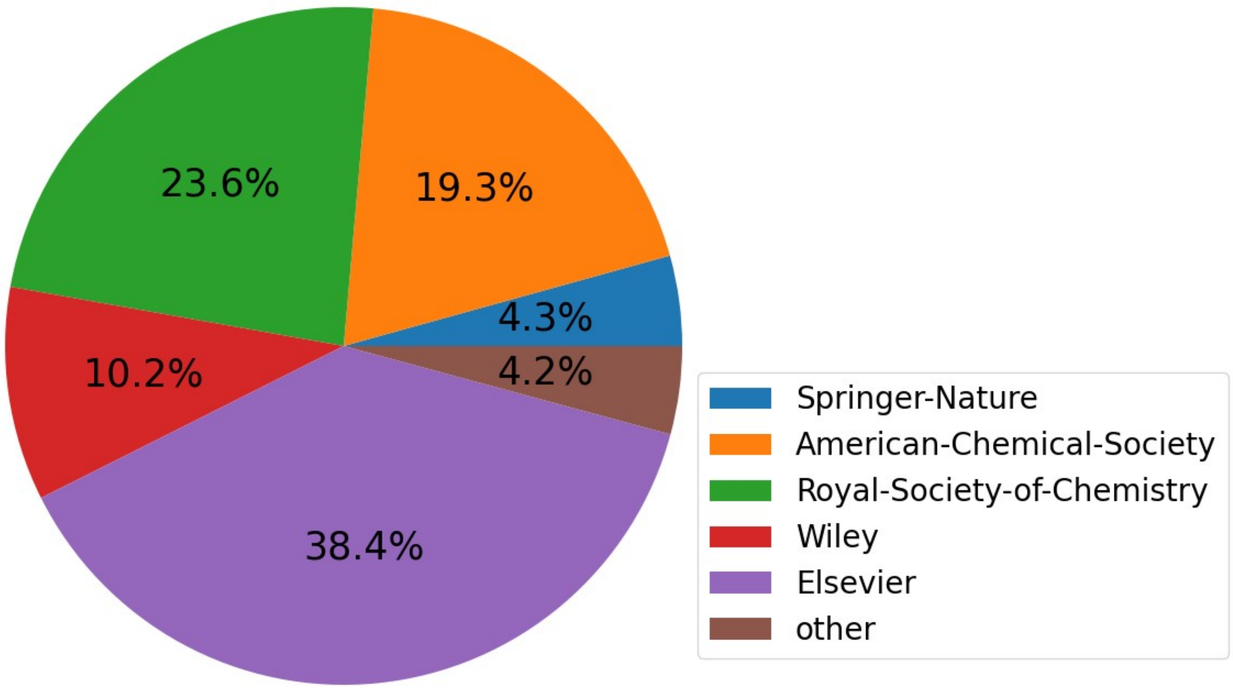

Supplementary Figure 1. Distribution of the papers for the major publishers of materials journals.

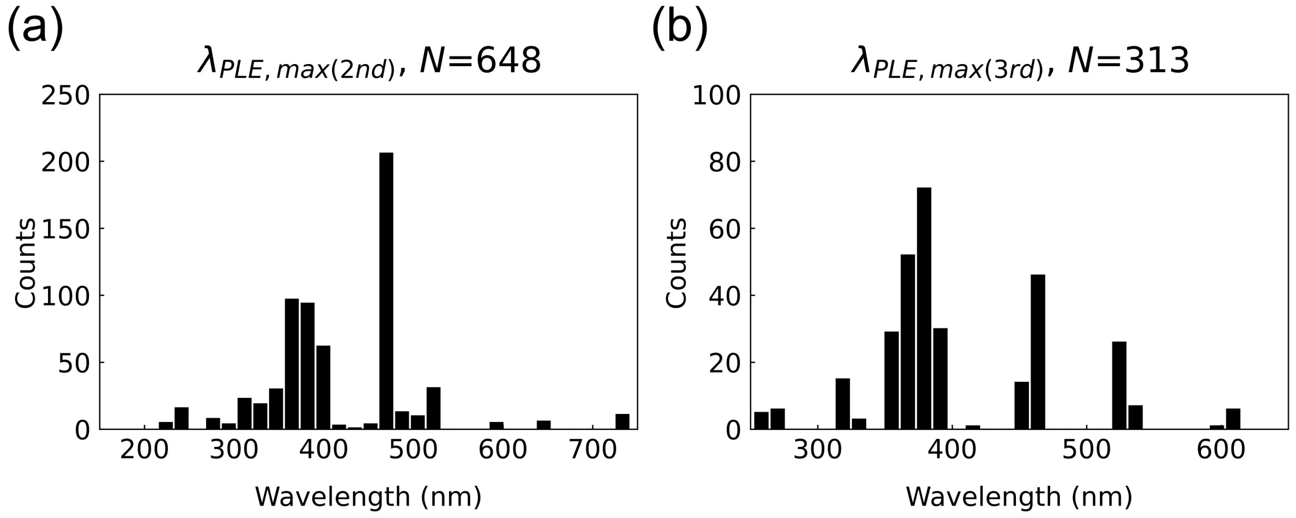

Supplementary Figure 2. Distribution information for $\lambda_{PLE,max(2nd)}$ and $\lambda_{PLE,max(3rd)}$.

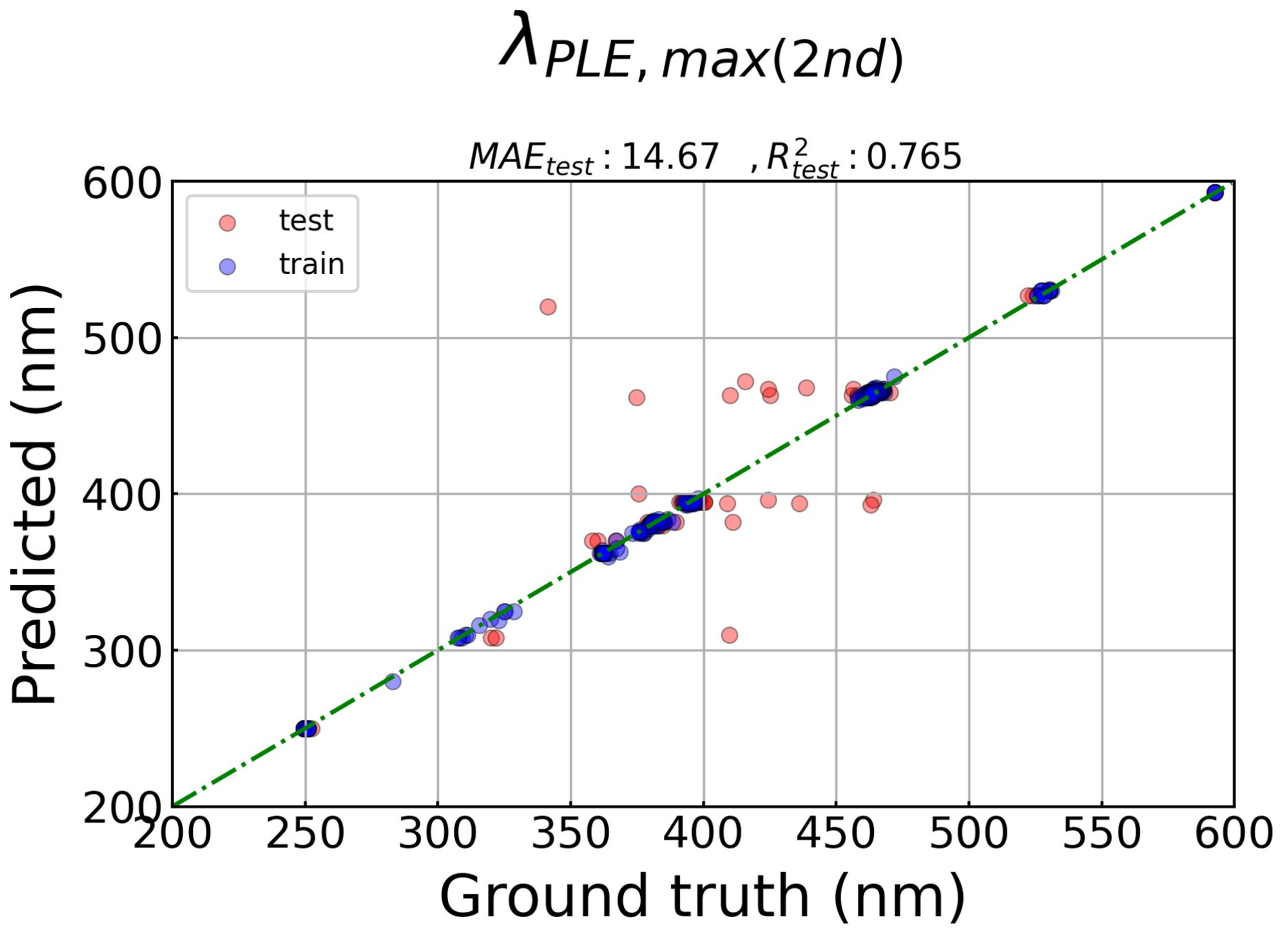

Supplementary Figure 3. Prediction result of the XGB method on $\lambda_{PLE,max(2nd)}$.

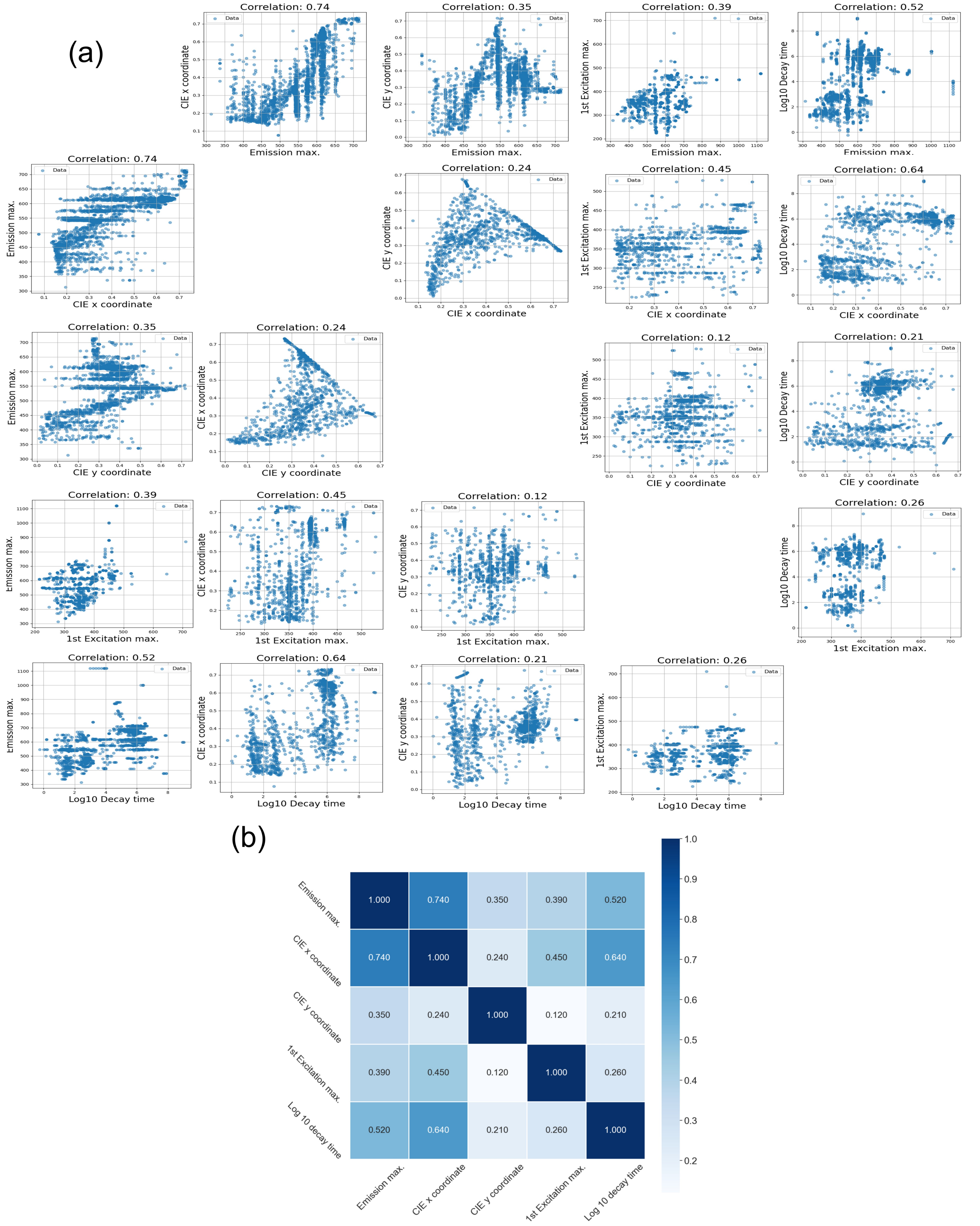

Supplementary Figure 4. Correlation between targets of inorganic phosphor database. (a) Scatter plots and (b) heatmap of the correlation between targets.

Target property	Temp. (T)	λ_{exc}	$\lambda_{PLE,dec}$
$\lambda_{PL,max}$	O	O	
X_{CIE}	O	O	
Y_{CIE}	O	O	
QE_{int}	O	O	
QE_{ext}	O	O	
T50		O	
$\lambda_{PLE,max}$	O		O
$\tau_{log,PL}$	O	O	O

Supplementary Table 1. Prediction results of inorganic phosphor properties according to various feature (measurement conditions) combinations.

Target property	$\lambda_{PL,max}$	$\lambda_{PLE,max(1st)}$	$\lambda_{PLE,max(2nd)}$	$\tau_{log,PL}$	X_{CIE}	Y_{CIE}	QE_{int}	QE_{ext}	$T50$
count	3760	1864	648	2211	2744	2744	1147	139	453
mean	573.11	368.20	417.11	4.55	0.42	0.35	46.15	38.35	479.25
std	90.62	54.17	82.62	1.91	0.18	0.12	25.19	22.11	84.30
min	313.00	215.00	215.00	-0.24	0.08	0.01	0.11	0.10	323.00
25%	535.00	336.00	364.00	2.67	0.27	0.29	27.05	20.40	420.00
50%	596.00	377.00	395.00	5.56	0.38	0.35	43.49	38.00	470.00
75%	616.00	395.00	465.00	6.09	0.61	0.40	65.75	53.70	537.00
max	1120.00	710.00	742.00	9.01	0.73	0.72	99.00	90.00	823.00

Supplementary Table 2. Descriptive statistics of the target properties of the inorganic phosphor dataset.
